# Choroidal thickness and vascular microstructure parameters in Chinese school-age children with high **hyperopia** using optical coherence tomography

**DOI:** 10.3389/fped.2023.1092153

**Published:** 2023-02-06

**Authors:** Dehai Zhu, Hui Wang, Ruoshi Li, Jing Wen, Ruiying Li, Jingjing Zhao

**Affiliations:** ^1^ Department of Pediatric Ophthalmology, Peking University First Hospital, Beijing, China; ^2^ Peking University Children Vision Institute, Beijing, China; ^3^ Department of Ophthalmology, Beijing Chaoyang Hospital, Capital Medical University, Beijing, China

**Keywords:** choroidal thickness, choroidal vascularity index, EDI-OCT choroidal thickness, EDI-OCT, high hyperopia

## Abstract

**Background:**

The current study was to evaluate the choroidal thickness (CT) and vascular microstructure parameters in Chinese children with high hyperopia through enhanced depth imaging optical coherence tomography (EDI-OCT).

**Methods:**

Cross-sectional study. A total of 23 children with high hyperopia and 29 children with normal refractive status were retrospectively enrolled in the study. The measurement of the macular CT, 7 points: the sub-foveal area point, the temporal and nasal points at a radius of 0.5-mm, 1.5-mm, and 3-mm were measured. After binarization of the OCT images, the total choroidal area (TCA), stromal area (SA) as well as the luminal area (LA) were identified and measured. The choroidal vascularity index (CVI) was defined as the ratio of LA to TCA. The independent *t*-test for normal distributions and Kruskal-Wallis tests for non-normal distributions were used to compare other parameters between groups. The Tamhane's T2 test was performed to adjust for multiple comparisons between groups within each analysis.

**Results:**

The subfoveal CT (SFCT) in the high hypermetropic group was significantly thicker than that in normal controls (309.22 ± 53.14 μm vs. 291.27 ± 38.27 μm; *P* = 0.019). At 0.5 mm, 1.5 mm, and 3.0 mm in diameter, the nasal choroidal sectors of the high hyperopia eyes were significantly thicker than that of the control (*P* < 0.05). There was significant difference in the choroidal vascular parameters. TCA and LA in the high hyperopia eyes was significantly larger than that of the normal control eyes (3078129.54 ± 448271.18 μm^2^ vs. 2765218.17 ± 317827.19 μm^2^, 1926819.54 ± 229817.56 μm^2^ vs. 1748817.18 ± 191827.98 μm^2^; *P* = 0.009, *P* = 0.011; Table 2). SA values were 1086287.55 ± 212712.11 um^2^ in the high hyperopia eyes and 999712.71 ± 209838.12 μm^2^ in the control eyes. The CVI and LA/SA ratio values were differed significantly in the two groups (*P* = 0.019, *P* = 0.030, respectively). AL was significantly correlated with SFCT (r = −0.325, *P* = 0.047), but not significantly correlated with other parameters. Spherical equivalent (SE) was significantly correlated with AL and SFCT (r = −0.711, r = 0.311; *P* = 0.001, *P* = 0.016), whereas no significant association between sphere and other parameters.

**Conclusion:**

The choroidal structure of the high hyperopia eyes was different from the normal control eyes. The thicker SFCT, higher LA, and TCA were characteristic of high hyperopia eyes. Choroidal blood flow may be decreased in amblyopic eyes. SFCT of high hyperopia children abnormally increased and correlated with shorter AL and higher SE. AL and SE affect choroidal structure and vascular density.

## 1. Background

### 1.1. Clinical significance: we need an accurate method to evaluate the severity of hyperopia

By birth, human beings are predominantly hyperopic, and as the age progresses, hyperopic eyeballs grow to become emmetropic or even myopic (1–4). For children with refractive errors, the most common type is hyperopia worldly. If left untreated, the hyperopia, especially moderate-to-high hyperopia, may develop a series of sequelae, such as amblyopia and strabismus (5–8). Therefore, early diagnosis and severity stratification of hyperopia is vital for physicians to select appropriate management strategies, thus minimizing relevant sequelae.

### 1.2. Current status: current parameters and limitations

The choroid comprises blood vessels, melanocytes, fibroblasts, resident immunocompetent cells, and supporting collagenous and elastic connective tissue. It plays a vital role in ocular physiology by oxygenating and nourishing avascular outer retinal layers, particularly photoreceptors and the optic nerve pre-laminar section of the optic nerve (9). It is reported that the choroid may play an essential role in the visually guided regulation of eye growth and the development of myopia and hyperopia (1–4), so several choroid-related parameters have been investigated to evaluate the severity degree of hyperopia. Choroidal thickness (CT) is widely used in the evaluation of choroid status, and its value is associated with a range of factors, including age, gender, and refractive errors (10–15). However, CT provides limited information related to the choroid's subtle structural changes.

### 1.3. Problems: C.T. could not measure the subtle changes of hyperopia, but choroidal vascularity index might be

As we know, in the pathological changes of myopia, the choroidal thinning associated with myopic axial elongation is primarily the result of reduced thickness of the stromal rather than the vascular component of the choroid (16). In contrast, hyperopia eyes have shorter axis and a thicker choroid, but it is still not clear whether the choroid thickens due to stromal or vascular component. So, we need the parameters to provide more subtle structural changes, which are not available in CT.

### 1.4. The rationale of CVI: OCT presents the subtle vascular structures which are related to hyperopia

With the development of optical coherence tomography (OCT), the measurement of subtle choroid structures becomes possible. Recently, a new quantitative index, choroidal vascularity index (CVI), defined as the ratio of blood vessels' luminal area (LA) to the total choroidal area (TCA), has been developed to evaluate the vascular structure of the choroid (17). As CVI, which mainly measures choroidal vascularity, may play a more important role in evaluating hyperopia severity. Most of the previous studies focused on using the CVI to assess the subtle choroid structural changes related to myopia or high myopia. Still, its applications in children with high hyperopia have not been fully documented, especially in the Chinese pediatric population.

### 1.5. Aim of the study

In this study, we used the enhanced depth imaging OCT (EDI-OCT) to measure CT and CVI metrics in Chinese children with high hyperopia and normal controls. We aimed to determine the value of CVI as a characteristic parameter to evaluate the severity of hyperopia.

## 2. Methods

### 2.1. Study population

The Institutional Review Board of our hospital approved this observational cross-sectional study, and written informed consent were obtained from all participants.

The participants were consecutively recruited in the Department of Pediatric Ophthalmology of our hospital from June 2021 to September 2021. The inclusion criteria were (1) age between 4 and 14 years, (2) best-corrected visual acuity (BCVA) ≥ 0.8, (3) intraocular pressure (IOP) < 21 mmHg, (4) normal anterior chamber angles, (5) normal optic nerve head (ONH) without glaucomatous changes, such as the neuro-retinal rim narrowing, cup-disc ratio increasing, and no retinal nerve ber layer abnormalities. The participants with the following criteria were excluded, (1) history of ocular or systemic diseases, including congenital cataract and glaucoma, hypertension, and diabetes, (2) previous intraocular or refractive surgery, (3) neurologic disease, or (4) other evidence of retinal pathology. The demographic data of all enrolled patients were collected for analysis.

### 2.2. Ophthalmologic examinations

All participants underwent comprehensive ophthalmic examinations to collect the following data, which were BCVA, spherical equivalent (SE), slit-lamp biomicroscopy, axial length (IOL Master; Carl Zeiss Meditec, Dublin, CA), corneal curvature and IOP. Retinoscopy measurements were performed under the cycloplegia. The visual acuity was measured with a standard logarithmic visual acuity chart, and the decimal visual acuity was converted to the logarithm of the minimal angle of resolution (logMAR) units.

### 2.3. EDI-OCT examinations of retinal and choroidal thickness

The choroidal area was obtained by the EDI-OCT (Spectralis, Heidelberg Engineering, Heidelberg, Germany) by one ophthalmologist with ten years of experience in OCT exam throughout the study to measure CT and choroid vascular parameters.

**Figure 1:**
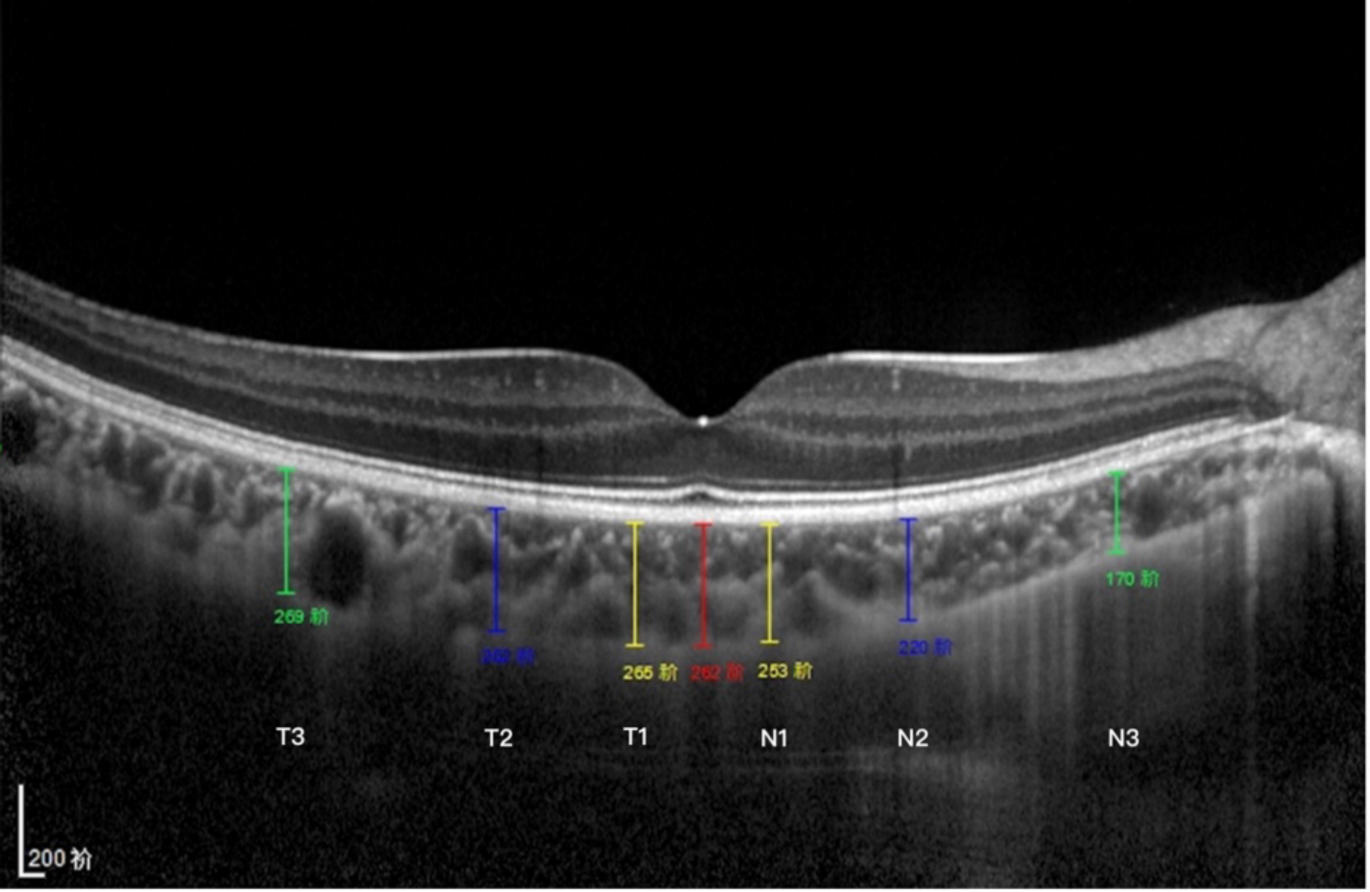
Subfoveal CT measurement. CT was measured at 7 points: directly beneath the fovea or the subfoveal area (SFCT) point, and 0.5/1.5/3 mm to the fovea nasally (N1/N2/N3), 0.5/1.5/3 mm to the fovea temporally (T1/T2/T3).

The macular CT was measured from the outer portion of the hyper-refractive line corresponding to the retinal pigment epithelium (RPE) to the inner surface of the sclera. The CT measurements were obtained at seven spots in a single horizontal scan centered on the fovea, including directly beneath the sub-foveal area CT (SFCT) point, the temporal and nasal points at a radius of 0.5- mm, 1.5-mm, and 3-mm (Figure 1). The elongation of AL may result in optical amplification effect. Littmann formula was used to calculate true image size to correct the optical amplification effect of OCT measurement.

### 2.4. Image binarization and choroidal vascularity index

**Figure 2:**
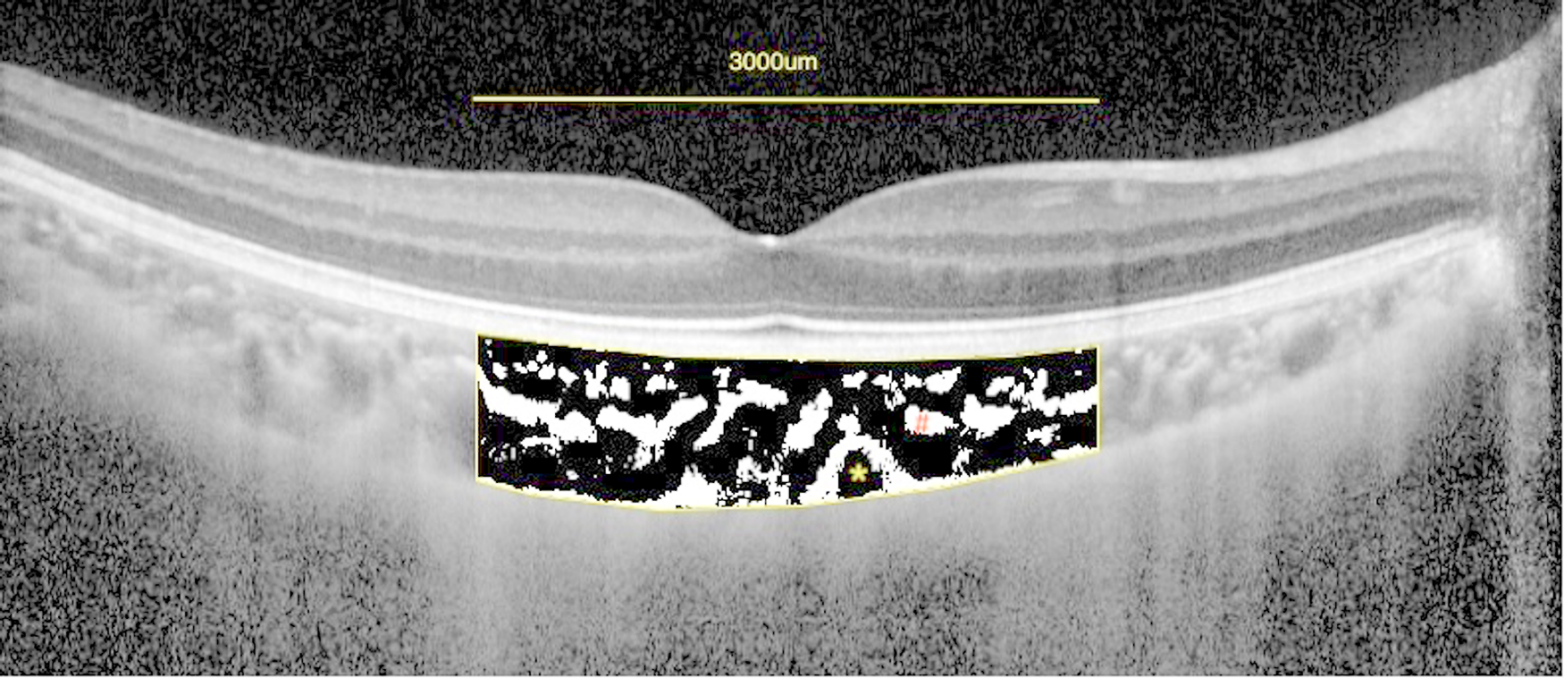
Enhanced depth imaging (EDI)-OCT image and converted binary image of a normal eye. An EDI-OCT image of a healthy eye (**A**) was converted to a binary image (**B**) using the ImageJ software. The luminal area (dark area, asterisk) and the interstitial area (cross) are seen. The rectangle surrounded by a red line was excised, and the dark areas were traced by the Niblack method.

Choroid vascular parameters were measured using the Niblack auto local threshold tool in the Image J platform (version 1.47, National Institutes of Health, Bethesda, MD, USA; http://imagej.nih.gov/ij/). Each segmented B-scan through the fovea on OCT was binarized with dark and light pixels to signify the L.A. and S.A. of the choroid, respectively (Figure 2). The TCA was calculated by multiplying the standard width of 3000 µm (1500 µm on the nasal and temporal side of the fovea) by the center choroidal thickness. Finally, TCA, LA, and SA were measured, followed by the CVI, defined as the ratio of LA to TCA.

**Figure 3:**
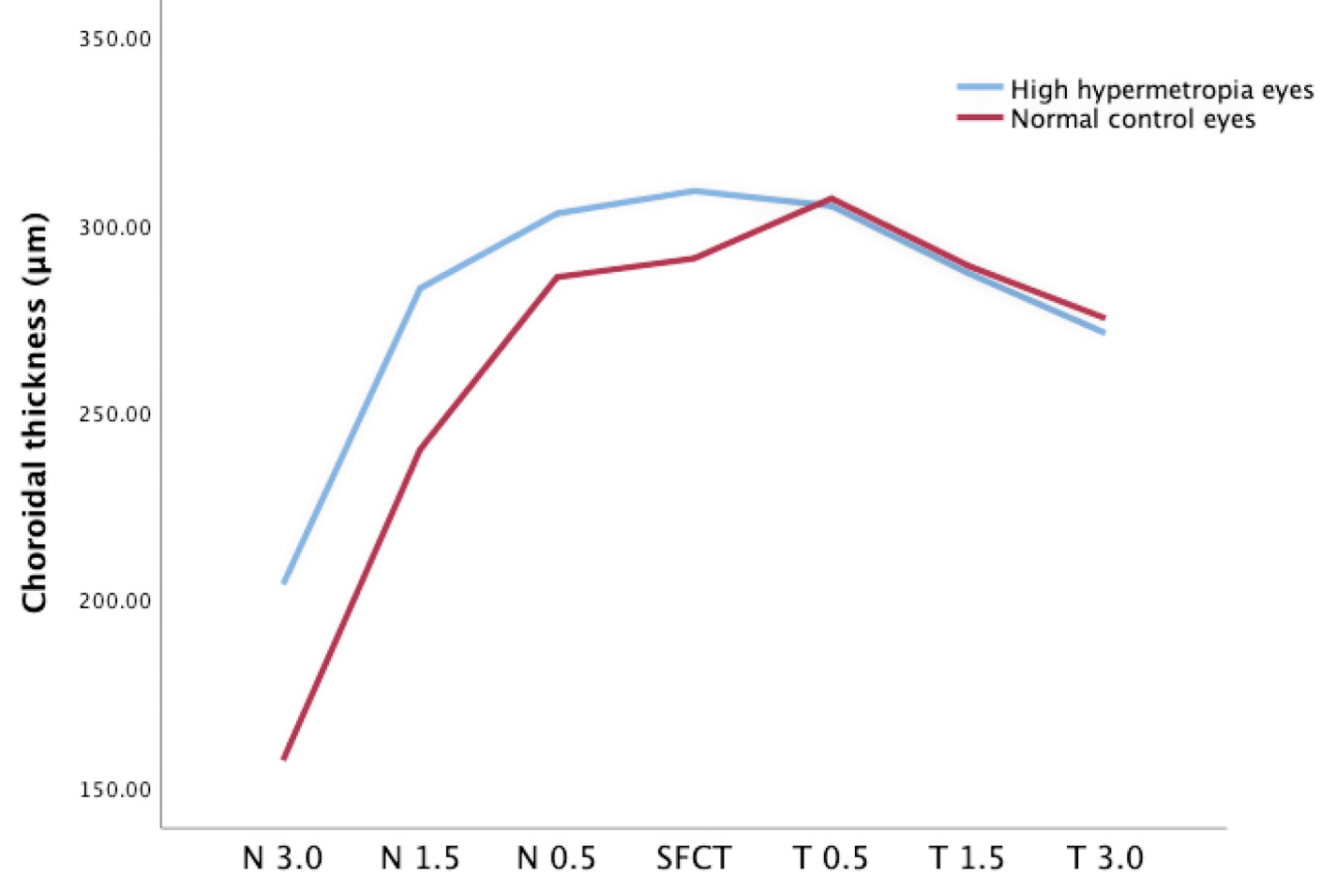
CT at different locations of high **hyperopia** and normal eyes.

### 2.5. Statistical analysis

The Kolmogorov-Smirnov test was used to analyze the normality of distribution. Descriptive statistics were calculated as the mean and standard deviation for normally distributed variables and median and interquartile range (IQR) for non-normally distributed variables. The independent *t*-test for normal distributions and Kruskal-Wallis tests for non-normal distributions were used to compare other parameters between groups. The categorical data were described as frequencies and percentages and compared between groups using Fisher's exact test as appropriate. The Tamhane's T2 test was performed to adjust for multiple comparisons between groups within each analysis. A two-sided *P* value less than 0.05 was considered statistically significant. Statistical analysis was performed using the SPSS software version 21 (SPSS, Inc., IL, USA).

Measures of central tendency in continuous data were described as median (or mean) and range, and categorical data were described as frequencies and percentages. The propensity score matching method was used to match the control cases with patients with complications. The continuous variables were compared using a *t*-test, and the ordinal variables were compared using conditional logistic regression. *P* < 0.05 was considered statistically significant.

## 3. Results

### 3.1. Image binarization and choroidal vascularity index

#### 3.1.1. Patients and baseline data

In the study interval, a total of **52** children were enrolled, including 23 with high hyperopia (female/male = 13/10, age 6.13 ± 2.24 years) and 29 normal controls (female/male = 15/14, age 7.00 ± 2.00 years) (Table 1). The patients were classified according to spherical equivalent (SE), which were high hyperopia with SE of + 5.00D or more, and normal controls with S.E. of −0.50D ∼ + 1.00D. The mean BCVA was 0.06 ± 0.11 logMAR units in the high hyperopia eyes and −0.01 ± 0.06 logMAR units in the control eyes.

**Table 1:** Demographic and clinical characteristics of enrolled participants (*n* = 52).

	High **hyperopia** group	Normal control group	*P* value
Number of eyes (*n*)	23	29	–
Age (year)	6.13 ± 2.24	7.00 ± 2.00	*P* = 0.152
Gender (male/female)	13/10	15/14	*P* = 0.801
SE (D)	+5.84 ± 1.97	0.24 ± 0.07	***P* < 0.001**
BCVA	0.06 ± 0.11	−0.01 ± 0.06	*P* = 0.504
IOP (mmHg)	16.85 ± 0.34	15.19 ± 0.61	*P* = 0.574
Axial length (mm)	20.55 ± 0.91	23.16 ± 0.45	***P* < 0.001**
Corneal curvature (mm)	8.02 ± 1.91	7.92 ± 1.69	*P* = 0.174

BCVA, best corrected visual acuity; D, diopter; SD, standard deviation; SE, spherical equivalent; logMAR, minimal angle of logarithm.

Factors with statistical significance are shown in bold.

The demographic and ocular characteristics were listed in the study (Table 1). Significant differences were observed on spherical equivalent (+5.84 ± 1.97D vs. 0.24 ± 0.07D, *P* < 0.001) and AL (20.55 ± 0.91 mm vs. 23.16 ± 0.45 mm, *P* < 0.001) between the two groups, while no differences were found on other parameters.

#### 3.1.2. Choroidal parameters measured by OCT

The SFCT was significantly thicker in the high hyperopia eyes than that in normal controls (309.22 ± 53.14 μm vs. 291.27 ± 38.27 μm, *P* = 0.017) (Table 2). At 0.5 mm, 1.5 mm, and 3.0 mm in diameter, the nasal choroidal sectors of the high hyperopia eyes were significantly thicker than that of the normal control eyes (*P* < 0.05). The temporal CT at 0.5 mm, 1.5 mm, and 3.0 mm were not significant different in high hyperopia eyes than that in normal control eyes (all *P* > 0.05) (Figure 3).

**Table 2:** Comparison of CT and choroidal vascular characteristics (*n* = 52).

	High **hyperopia** group (*n* = 23)	Normal control group (*n* = 29)	*P* value
**CT (um), mean ± SD**			
Sub-foveal	309.22 ± 53.14	291.27 ± 38.27	***P* = 0.017**
Nasal 0.5	303.21 ± 53.25	286.12 ± 41.81	***P* = 0.044**
Nasal 1.5	282.97 ± 40.82	239.75 ± 50.47	***P* < 0.001**
Nasal 3.0	204.98 ± 41.98	157.33 ± 35.40	***P* < 0.001**
Temporal 0.5	305.21 ± 43.22	307.01 ± 48.02	*P* = 0.145
Temporal 1.5	287.20 ± 41.28	288.80 ± 36.31	*P* = 0.471
Temporal 3.0	270.94 ± 35.91	275.55 ± 39.17	*P* = 0.386
**Choroid vascular parameters**			
TCA in mm^2^, mean ± SD	3078129.54 ± 448271.18	2765218.17 ± 317827.19	***P* = 0.009**
LA in mm^2^, mean ± SD	1926819.54 ± 229817.56	1748817.18 ± 191827.98	***P* = 0.011**
SA in mm^2^, mean ± SD	1086287.55 ± 212712.11	999712.71 ± 209838.12	*P* = 0.311
CVI (LA/TCA)	0.63 ± 0.06	0.64 ± 0.03	***P* = 0.019**
Luminal/stromal ratio	1.78 ± 0.26	1.74 ± 0.24	***P* = 0.030**

CT, choroidal thickness; CVI, choroidal vascularity index; CT, choroidal thickness; SA, stromal area; LA, luminal area; TCA, total choroidal area.

Factors with statistical significance are shown in bold.

There was significant difference in the choroidal vascular parameters. TCA and LA in the high hyperopia eyes was significantly larger than that of the normal control eyes (3078129.54 ± 448271.18 μm^2^ vs. 2765218.17 ± 317827.19 μm^2^, 1926819.54 ± 229817.56 μm^2^ vs. 1748817.18 ± 191827.98 μm^2^; *P* = 0.009, *P* = 0.011; Table 2). SA values were 1086287.55 ± 212712.11 μm^2^ in the high hyperopia eyes and 999712.71 ± 209838.12 μm^2^ in the control eyes. The CVI and LA/SA ratio values were differed significantly in the two groups (*P* = 0.019, *P* = 0.030, respectively).

#### 3.1.3. Relationship between choroidal parameters and their potential impact factors

Correlation analyses were performed between the choroidal parameters and age, AL, and SE (Table 3). Our results showed that AL was significantly correlated with SFCT and LA (r = −0.325, r = −0.417, *P* = 0.007), but not significantly correlated with other parameters. SE was found to be significantly correlated with AL and SFCT (r = −0.711, r = 0.311; *P* = 0.001, *P* = 0.016), whereas no significant association between sphere and other parameters.

**Table 3:** Correlation between choroid microstructure parameters and other baseline parameters in high hyperopia patients **.**

Parameters	AL		SFCT		LA		SA		TCA		CVI	
	r	*P*	r	*P*	r	*P*	r	*P*	r	*P*	r	*P*
Age	0.128	0.465	−0.220	0.204	−0.159	0.363	−0.198	0.255	−0.163	0.349	0.005	0.975
AL	–	–	−0.325	**0.047**	−0.417	**0.007**	−0.218	0.208	−0.254	0.141	0.071	0.687
SE	−0.711	**0.001**	0.311	**0.016**	−0.079	0.651	−0.156	0.372	−0.098	0.576	0.076	0.666

AL, axial length; SFCT, sub-foveal choroidal thickness; S.A., stromal area; SE, spherical equivalent; L.A., luminal area; TCA, total choroidal area; CVI, choroidal vascularity index.

Factors with statistical significance are shown in bold.

## 4. Discussion

This study has provided a comprehensive assessment of the topographical variations in choroidal microstructure parameters in Chinese children with high hyperopia. CT is different between the two groups and the significantly higher in the high hyperopia group. A significant difference was found in the following spots, including sub-foveal, Nasal 0.5, Nasal 1.5, and Nasal 3.0. Our analyses also showed that TCA, LA, and the LA/SA ratio of high hyperopia eyes were significantly larger, CVI was significantly smaller than that of the normal control eyes.

CT is the main parameter used to obtain information about the choroidal layer and has been examined in children and adolescents in many studies (18–21). In healthy pediatric populations, the mean sub-foveal from these studies ranged from 245 to 361 μm (18, 20, 21). Read et al. (22) reported that the mean of the sub-foveal CT was 330 ± 65 μm in Australian children (4–12 years) with spherical equivalent refractive errors (SER) (+1.25–−0.50 D). Takafumi Mori and Yukinori Sugano also found a similar result in preschool Japanese children (23). Additionally, Zha Yi et al. (24) reported that the SFCT was of 328.12 ± 65.93 μm in 4–12 years old Chinese children with SER was between −1.75D to +0.63D. Our study in normal children had very close values for age-matched normal children. The value of CT in evaluating the degree of refractive errors or amblyopia had also been depicted in some studies (19, 23–25). The results are roughly the same in different cohorts: the myopic eyes had thinner sub-foveal choroidal thickness, and the hyperopic eyes and hyperopic amblyopic eyes had thicker CT than emmetropic eyes and myopic eyes. However, few studies focused on the structural characteristic of the choroid in high hyperopia subjects. In the current study, both the subfoveal CT and topographical variation in high hyperopic children were described. Consistent with the existing conclusions, we found that the SFCT in high hyperopia children was significantly thicker than that in the normal control group. The average CT in the hyperopia group in our study was similar to the results of previous studies, but they seemed to be lower than those in the hyperopic amblyopia children with lower SER, which might verify the previous speculation that hyperopia was associated with subfoveal ChT, whereas amblyopia had no significant independent effect on subfoveal ChT in our study population (24). The pathophysiologic mechanisms involved need further verification. Additionally, the topographic features of the choroid in the hyperopia group in this study were almost the same as those in controls: the choroid was the thickest in the sub-foveal region and the thinnest in nasal regions which approach the optic nerve. These findings were also consistent with previous results of children either with different refractive status or with amblyopia, indicating the relatively stable choroid contour in pediatric populations (19, 24).

In normal eyes, CT in children can be affected by various factors, such as age, gender, diopter, and axial length. Previous studies reported a significant association between the AL and CT (26–28). Nagasawa et al. and Bidaut-Garnier reported that CT was negatively correlated with AL (29, 30). Li et al. reported that a thinner subfoveal CT correlated with a longer AL but no other factors, such as age, sex, and SER in myopic children (31). Read et al. found that CT increased in children with normal axial elongation, whereas children undergoing faster AL elongation tended to exhibit less thickening and a thinning of the choroid in some cases (32). In the current study, a thinner SFCT was also found to be negatively correlated with longer A.L. However, the causal relationship between choroidal thickening and hyperopia remains controversial.

OCT system uses fixed eye AL to scan. With the elongation of AL, the scanning area increases, resulting in optical amplification effect. Even if the Littmann formula is used to correct the optical amplification effect, there will be some errors in the results. CT could be also affected by sorts of variables and could not represent the choroid's subtle structural changes; we cannot assess the choroid structural changes and blood supply only by CT. measurement. Recently, Agrawal et al. (17) assessed the LA, SA, and TCA through EDI-OCT images and proposed a new parameter-CVI to assess the choroidal vascular structure. In their study, they found that CVI was less affected than the sub-foveal CT and suggested CVI be a more robust marker of choroidal diseases. Subsequently, other studies have also demonstrated less variability of CVI.

However, there are conflicting views on choroidal blood flow in the study of choroidal hyperopic patients. Baek et al. (33) claimed that hyperopia eyes were found to have increased choroidal CV compared to normal eyes. But most studies concluded that the choroidal blood flow in patients with high hyperopia eyes is lower than that in normal children (34–36). The results in this study are consistent with most of the previous studies, the average CVI in the hyperopic amblyopic eyes was lower than the normal control eyes. In our study, we found that the AL was significantly correlated with SFCT but not the CVI, which was consistent with the previous results, further indicating that CVI might be an alternative metric for the evaluation of choroidal disorders.

On the other hand, a shorter AL tended to be associated with bigger LA, SA, and TCA. Li et al. reported that changes in luminal areas might directly influence choroidal thickness, as blood vessels represent the main component of the choroid (31). Alis et al. (37) found that TCA and SA were higher in hyperopia than in both emmetropic and myopic eyes. Nishi et al. found that the LA was significantly larger, the SA was significantly smaller, and the luminal/stromal ratio was larger in amblyopic eyes than that of normal children (38). Meryem et al. (37) found that TCA and SA were higher in hyperopia than in both emmetropic and myopic eyes. In the present study, LA, and TCA values of the high hyperopia eyes were significantly larger than that of the normal control eyes suggesting an increase in blood flow and blood vessel area in eyes with shorter AL. It is still unknown whether the thickening of the choroid in hyperopia is caused by the increase of stromal area or the enlargement of the vascular luminal area. Multicenter studies with large sample sizes are needed for further validation.

Age is also an important factor affecting choroid structure. Previous studies found that the CT tended to decrease with increasing age in adults (27, 28), Fujiwara et al. found that choroidal vascular density had a negative association with age in healthy adults (39). In the present study, SFCT had a significant negative relationship with AL and positive relationship with SE. Ruiz-Medrano et al. (40) found that the choroidal stromal area was not affected by age in a healthy population that included normal children. In our study, there were no significant association among AL, SA, LA, and TCA.

Our study has several limitations. First, the sample size is relatively small, and the high hyperopes are all Chinese children. Second, the OCT measurement of each subject in our study was performed at a randomized time at their convenience, and the CT measurements only occurred in a single horizontal scan centered on the fovea. Further studies with a large population and unified examination time are needed for more positive and robust results.

## 5. Conclusions

In summary, to our knowledge, the current study provides the first description of the structural features of the choroid and the factors influencing them in Chinese children with high hyperopia.

The choroidal structure of the high hyperopia eyes was different from the normal control eyes. The thicker SFCT, higher LA, and TCA were characteristic of high hyperopia eyes. Choroidal blood flow may be decreased in amblyopic eyes. SFCT of high hyperopia children abnormally increased and correlated with shorter AL and higher SE. AL and SE affect choroidal structure and vascular density. We suggest that choroidal topographic features, are biomarker of high hyperopia, but CVI is another more robust indicator.

## Data Availability

The raw data supporting the conclusions of this article will be made available by the authors, without undue reservation.
